# Blended learning program for the development of skills in the aspiration of artificial airways*

**DOI:** 10.1590/1518-8345.4539.3462

**Published:** 2021-08-30

**Authors:** Aldenora Laísa Paiva de Carvalho Cordeiro, Fernanda Titareli Merizio Martins Braga, Luciana Regina Ferreira da Mata, Karina Dal Sasso Mendes, Rafael Cordeiro Fófano, Maria Célia Barcellos Dalri

**Affiliations:** 1 Universidade Federal do Triângulo Mineiro, Hospital de Clínicas, Gerência de Ensino e Pesquisa, Uberaba, MG, Brazil.; 2 Universidade de São Paulo, Escola de Enfermagem de Ribeirão Preto, PAHO/WHO Collaborating Centre for Nursing Research Development, Ribeirão Preto, SP, Brazil.; 3 Universidade Federal de Minas Gerais, Escola de Enfermagem, Belo Horizonte, MG, Brazil.; 4 Polícia Militar de Minas Gerais, Corpo Aéreo, Uberlândia, MG, Brazil.

**Keywords:** Nursing Process, Nursing Education, Educational Technology, Suction, Artificial Respiration, Validation Study, Processo de Enfermagem, Educação em Enfermagem, Tecnologia Educacional, Sucção, Respiração Artificial, Estudos de Validação, Proceso de Enfermería, Educación en Enfermería, Tecnología Educacional, Succión, Respiración Artificial, Estudio de Validación

## Abstract

**Objective:**

to develop and validate a blended learning program, of the inverted classroom type, on the aspiration of artificial airways.

**Method:**

applied and methodological research that involved technological production for teaching a Nursing Intervention, based on Vygotsky’s theoretical frameworks and on the Nursing Process. For elaboration and validation, a classic instructional design model was followed. The general and pedagogical requirements were validated, as well as those for videos and interface. For the analysis, the Content Validity Index and the First-order agreement coefficient were used.

**Results:**

34 experts participated, 27 of whom were nurses and seven were professionals in Information Technology. In the nurses’ opinion, the general and pedagogical requirements obtained a Content Validity Index of 0.99 and 0.98 was obtained for the videos and for the interface. The interface for the IT professionals was 0.94. All requirements showed almost perfect agreement.

**Conclusion:**

the teaching program was elaborated and validated by experts and constitutes an innovative proposal to train nurses. The mediation of teaching by means of duly validated technologies can favor learning and reaching positive results in the development of skills in the practice of aspiration of artificial airways.

## Abstract (pt)

**Objetivo:**

elaborar e validar um programa de ensino híbrido, do tipo sala de aula invertida, sobre a aspiração de vias aéreas artificiais.

**Método:**

pesquisa aplicada e metodológica que envolveu produção tecnológica para o ensino de uma Intervenção de Enfermagem, alicerçada nos referenciais teóricos de Vygotsky e no Processo de Enfermagem. Para elaboração e validação, seguiu-se um modelo de *design* instrucional clássico. Foram validados os requisitos gerais e pedagógicos, requisitos para vídeos e interface. Para a análise, foram utilizados o Índice de Validade de Conteúdo e o *First-order agreement coefficient*.

**Resultados:**

participaram 34 peritos, sendo 27 profissionais enfermeiros e sete profissionais da Tecnologia de Informação. Na visão dos enfermeiros, os requisitos gerais e pedagógicos obtiveram um Índice de Validade de Conteúdo de 0,99; para os vídeos e para a interface obteve-se 0,98. A interface para os profissionais da informática foi de 0,94. Todos requisitos apresentaram concordância quase perfeita.

**Conclusão:**

o programa de ensino foi elaborado e validado por peritos e constitui-se uma proposta inovadora para formar enfermeiros. A mediação do ensino por meio de tecnologias devidamente validadas pode favorecer o aprendizado e a obtenção de resultados positivos no desenvolvimento de habilidades da prática da aspiração de vias aéreas artificiais.

## Abstract (es)

**Objetivo:**

desarrollar y validar un programa educativo híbrido, similar al aula invertida, sobre la aspiración de vías aéreas artificiales.

**Método:**

investigación aplicada y metodológica que involucró producción tecnológica para la enseñanza de una Intervención de Enfermería, basada en los marcos teóricos de Vygotsky y el Proceso de Enfermería. Para la elaboración y validación se siguió un modelo clásico de *design* instruccional. Se validaron los requisitos generales y pedagógicos, requisitos para videos e interfaz. Para el análisis se utilizó el Índice de Validez de Contenido y el *First-order agreement coefficient*.

**Resultados:**

participaron 34 expertos, de los cuales 27 eran enfermeros y siete profesionales en Tecnologías de la Información. Según la perspectiva de los enfermeros, los requisitos generales y pedagógicos obtuvieron un Índice de Validez de Contenido de 0,99; para los vídeos y para la interfaz se obtuvo 0,98. La interfaz para los profesionales de la informática fue de 0,94. Todos los requisitos mostraron una conformidad casi perfecta.

**Conclusión:**

el programa educativo fue diseñado y validado por expertos y constituye una propuesta innovadora para la formación de enfermeros. La mediación de la enseñanza a través de tecnologías debidamente validadas puede favorecer el aprendizaje y la obtención de resultados positivos en el desarrollo de habilidades en la práctica de la aspiración de vías aéreas artificiales.

## Introduction

The centrality of Nursing is care for the individual, family or human community and the Nursing Process (NP) is the way to give it scientific rationality capable of enhancing knowledge and the professional practice^(1)^. The NP consists of the attitude of scientific doing in Nursing. As the nurses learn more about this way of doing Nursing, they note that the care provided has particularities and is materialized by resolute actions, based on the most innovative care practices.

The NP has five stages: research, nursing diagnosis, planning, implementation and evaluation. Such stages are capable of providing subsidies for the nurse’s clinical reasoning. In the planning stage, after making a diagnostic decision, the nurse needs to select the best interventions, always considering obtaining positive results^(2)^.

The “aspiration of artificial airways” Nursing Intervention (NI) is an expanded approach that consists of removing secretions from the lower respiratory tract through a device with a negative pressure system, with aseptic technique and with the purpose of maintaining pervious airways^(3)^. In order to accurately detect the clinical signs that determine treatment with aspiration of artificial airways and perform it free of harms, it is necessary to continuously train Nursing professionals to effectively carry out this intervention^(4)^.

In 2017, the Federal Nursing Council (*Conselho Federal de Enfermagem*, COFEN) published resolution 557/2017^(5)^, which standardized the role of Nursing in airway aspiration, demanding from nurses a proactive attitude in view of the obligation to investigate and correctly identify the need for this intervention, in addition to implementing it safely and developing a practice based on ethical and human precepts, supported by the best scientific evidence, with results monitoring.

The availability of evidence on artificial airway aspiration (AAA) still presents challenges for the professionals who perform it. In addition, the guidelines available in the scientific literature are not yet implemented in the clinical practice^(6)^.

To guarantee the quality of the teaching-learning process and personalized Nursing care for patients with specific needs, it is necessary to structure forms of education based on the results of clinical and educational scientific investigations^(7)^.

The teaching and learning process mediated by Information and Communication Technologies (ICT) seeks an innovative educational landscape employing changes in the way teachers teach courses and disciplines. In this sense, the blended learning modality -in-person and mediated by digital technology- stands out, with emphasis on the inverted classroom, which has been evidenced as an approach capable of improving student performance, as well as the perceptions of the learning experience^(8-9)^.

Blended learning emerges as a promising approach to education in Nursing. In this conception, students have a variety of possibilities in the use of technology and countless communication practicalities about knowledge, making them motivated, involved in the learning process, committed and dedicated in their studies^(10)^.

With the advancement of the ICT in education, the development and validation of blended learning programs in Nursing are increasingly considered opportune. In view of the gap in the literature regarding the existence of studies that contemplate educational technologies valid for the teaching and learning of this theme, the present study aimed to develop and validate a blended learning program, of the inverted classroom type, on AAA.

## Method

This is an applied and methodological research study, which involved technological production for teaching a NI, based on the Vygotsky’s theoretical frameworks^(11)^ and on the NP. The guidelines of the Revised Standards for Quality Improvement Reporting Excellence (SQUIRE 2.0) were adopted, as it is a study to improve quality of care, through the proposal of a teaching program.

For the elaboration and validation of the teaching program proposed in this study, the classic instructional design model ADDIE was used, which is an acronym for *a*nalyze, *d*esign, *d*evelop, *i*mplement and *e*valuate. These stages form a dynamic, flexible, effective and efficient guide^(12)^.

Instructional design analysis seeks to understand the educational problem and its possible solution^(12)^. This phase consisted of conducting a literature review, which sought to broaden the understanding of AAA and subsidize the elaboration of the teaching plan, of Learning Objects (LO) -digital resources that can be used, reused or referenced during the technology-supported learning^(12)^- and of assessment tools.

The main findings of the literature review were the clinical signs that indicate the need for AAA, the recommendations for execution and its main complications. Based on these findings, the teaching plan was elaborated, covering research, diagnostic and therapeutic reasoning and the assessment of AAA through LO, which included videos and infographics.

The design referred to the planning moment in which the target audience, the necessary workload for the educational action, the learning objectives, the available resources and the evaluation of learning and of the references were defined.

In the development phase, the LO (two videos, two infographics and a virtual library) and the instruments for the assessment of learning (multiple choice questions and the Objective Structured Clinical Examination – OSCE) were developed.

In the ADDIE instructional design, the implementation is subdivided into two phases: publication and execution^(12)^. The first consists of making the learning units available to the experts and the second is the moment when the students carry out the activities proposed by the teaching program^(12)^.

In this study, it was chosen to host this teaching program through the Virtual Learning Environment (VLE) called Modular Object-Oriented Dynamic Learning Environment (Moodle) of the Extension Courses of the University of São Paulo. The inclusion of content, LO and assessment instruments was carried out by the researchers in the months of December 2019 and January 2020.

**Figure 1:**
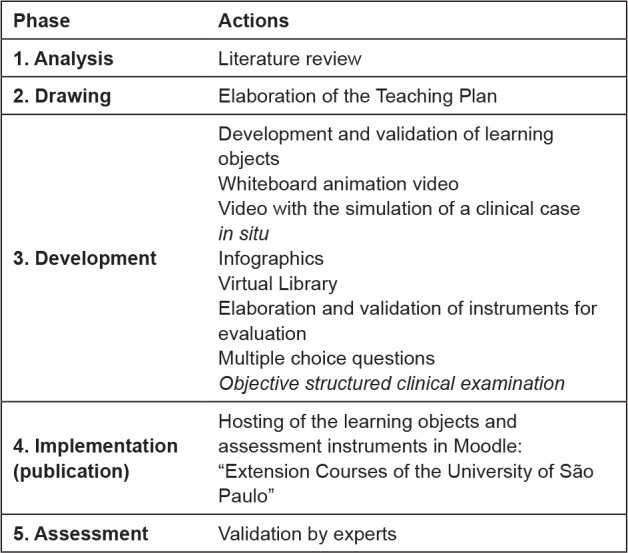
Phases of the instructional design that were covered and the actions taken in this study. Ribeirão Preto, SP, Brazil, 2020

Figure 1 shows the phases of the ADDIE instructional design covered in this study.

For the validation of the teaching program, the population was made up of Nursing and Information Technology (IT) professionals.

The criteria proposed by a scholar^(13)^ for the selection of Nursing experts were adopted, considering a minimum score of five points to be included in the study. For the IT area, the following inclusion criteria were chosen: experience with technical support, programming or networking. Those participants who did not return the instrument within 12 days were excluded.

The search for experts took place through the Lattes Platform from the National Research Council (CNPQ) of Brazil, in research groups related to the theme of this study. Invitations were sent via e-mail, setting out the objectives, to 35 Nursing professionals and ten IT professionals in January 2020. The number of invitations sent to each professional category was motivated due to the requirements to be validated for the teaching program. The IT professionals only participated in interface validation, while the Nursing professionals validated all requirements.

Those who agreed to participate were sent, at the end of the invitation, a link that directed them to a Google Forms type form, with the Free and Informed Consent Form (FICF) and, subsequently, to the instruments for evaluation, with the relevant explanations and the link where the teaching program was hosted, in order to allow the experts to assess it. Access was allowed through a visitor password. For those who clicked “I do not accept to participate in this study”, the document was closed.

For the characterization of the Nursing experts, the following variables were used: gender, age, area of performance, highest academic degree, area of highest academic degree, years of professional experience, articles published in the last two years, participation in scientific events, participation in some distance or blended course and development or participation in the development of some LO.

The IT expert was characterized by the gender, age, academic background, area of performance, academic degree, participation in some distance or blended course and development or participation in the development of some LO variables.

The instrument used for the validation of the teaching program was adapted from an existing instrument in the literature^(14)^, with authorization. The general/pedagogical requirements and those specific to the videos and interface were considered. For the IT experts, only the interface requirements of the teaching program were sent.

The data were coded, categorized and typed (double entry) in a Microsoft Excel^®^ spreadsheet. Subsequently, they were transferred and processed using the Statistical Package for the Social Sciences (SPSS), version 20.0. The variables for the characterization of the experts were analyzed according to descriptive statistics, through the distribution of absolute and percentage frequency and measures of centrality (mean) and dispersion (standard deviation, minimum and maximum values). The Content Validity Index (CVI) was calculated for each requirement. Gwet’s First-order Agreement Coefficient (AC1) was used to verify the reliability of the agreement between the experts. The program used for the agreement analyses was R, version 3.6.2, from the irrCAC library. The preference for using Gwet’s AC1 over Cohen’s kappa coefficient was due to the fact that the first is a more stable reliability coefficient than the second, that is, less affected by substantially unbalanced marginal totals^(15)^. For the classification of agreement, poor was adopted for Gwet’s AC1 below 0.00; light, for values between 0.00 and 0.20; acceptable, between 0.21 and 0.40; moderate, between 0.41 and 0.60; considerable, between 0.61 and 0.80; and almost perfect, between 0.81 and 1.00^(16)^. The CVI considered acceptable was at least 0.80 and, preferably, greater than 0.90^(17)^.

The project followed the legal procedures determined by Resolution 466/2012 of the National Health Council (*Conselho Nacional de Saúde*, CNS), with regard to research involving human beings. It was submitted to the appreciation of a Research Ethics Committee, under CAAE number 95152918.8.0000.5393, with the issuance of a favorable opinion on September 27^th^, 2018.

## Results

The theoretical content mediated by digital technology of the teaching program was organized into six topics: welcome and information about the teaching program, initial test, introduction to AAA, AAA through an open system, virtual library and intermediate and final tests. A topic was added to provide information to the expert about the face-to-face moment: skills training, simulation scenarios and OSCE.

The sample of this validation corresponded to 34 experts, them being 27 professionals from the Nursing area and seven from IT.

The mean age of the Nursing experts was 37 years old, with a standard deviation of 6.1, a minimum age of 29 years old and a maximum of 57. The female gender was predominant, with 23 (85.2%) experts. As for the length of professional experience, the mean was 12.9 years, with a standard deviation of 6.7, a minimum of one year and a maximum of 35. The predominant professional practice area was teaching in Nursing, with 14 (51.9%) experts, as shown in Table 1.

**Table 1:** Characterization of the Nursing experts (n = 27) for the validation of the Teaching Program. Ribeirão Preto, SP, Brazil, 2020

Variable	N	%
**Professional performance** Teaching	14	51.9
Intensive care	4	14.8
Urgency and emergency	3	11.1
Permanent education	2	7.4
Medical clinic	2	7.4
Surgical Clinic	1	3.7
Obstetric urgencies emergencies	1	3.7
**Total**	**27**	**100**
**Highest academic degree** Master’s	10	37
PhD	17	63
Total	27	100
**Variable**	**N**	**%**
**Area of the highest academic degree** Health sciences	15	55.6
Health care	8	29.6
Nursing	3	11.1
Urgency and emergency	1	3.7
Total	27	100
**Participation in a scientific event in the area of performance in the last 2 years** Yes	23	85.2
No	4	14.8
Total	27	100
**Publication of a scientific article in the field of performance in the last 2 years** Yes	19	70.4
No	8	29.6
Total	27	100
**Participation in distance or blended course** Yes	26	96.3
No	1	3.7
Total	27	100
**Learning Object Development** Yes	14	51.9
No	13	48.1
**Total**	**27**	**100**

The mean age of the IT experts was 33.3 years old, standard deviation of 5.6, minimum age of 29 years old and maximum of 44. The male gender was predominant, with six (85.7%) experts. As for the length of professional experience, the mean was 10.4 years, with a standard deviation of 3.9, a minimum of five years and a maximum of 16. Table 2 shows the characterization of the IT experts.

**Table 2:** Characterization of the experts in the Information Technology area (n = 7) for the validation of the Teaching Program interface. Ribeirão Preto, SP, Brazil, 2020

Variable	N	%
**Academic training** Systems Analysis and Development	4	57.1
Information Systems	2	28.6
IT	1	14.3
Total	7	100
**Area of professional practice** Programming	3	42.9
Technical Support	3	42.9
Network Management	1	14.3
Total	7	100
**Academic degree** Specialization	5	71.4
Graduation	2	28.6
Total	7	100
**Area of the academic degree** Systems Analysis and Development	2	28.6
Web Development	1	14.3
Development of Systems for Mobile Applications	1	14.3
Development of Business Management Systems	1	14.3
Information Systems	1	14.3
Networks	1	14.3
Total	7	100
**Participation in distance or blended course** Yes	6	85.7
No	1	14.3
Total	7	100
**Learning Object Development** Yes	1	14.3
No	6	85.7
**Total**	7	100

Table 3 presents the validations of the pedagogical requirements and specific requirements for videos and interfaces, performed by the Nursing experts. Table 4 presents the interface validation by the IT experts.

**Table 3:** Validation of general and pedagogical requirements, specific requirements for videos and interface of the Teaching Program by experts in the field of Nursing (n = 27). Ribeirão Preto, SP, Brazil, 2020

Agreement Criterion	Strongly disagree	Disagree	Agree	Strongly agree	Don't know	CVI*
	n (%)	n (%)	n (%)	n (%)	n (%)	
**General and pedagogical requirements** Educational environment	-	-	7 (25.9)	20 (74.1)	-	1.00
Relevance to the teaching plan	-	-	7 (25.9)	20 (74.1)	-	1.00
Ease of use of the motivational aspects and respect for the individualities	-	-	12 (44.4)	13 (48.1)	2 (7.4)	1.00
Clarity of the contents	-	-	6 (22.2)	21 (77.8)	-	1.00
Correction of the contents	-	-	6 (22.2)	21 (77.8)	-	1.00
Vocabulary adaptation according to the target audience	-	-	6 (22.2)	21 (77.8)	-	1.00
Informational load	-	-	7 (25.9)	20 (74.1)	-	1.00
Integration of the learning objects	-	-	6 (22.2)	21 (77.8)	-	1.00
Error management	-	-	12 (44.4)	12 (44.4)	3 (11.1)	1.00
Easy to memorize	-	-	6 (22.2)	20 (74.1)	1 (3.7)	1.00
User documentation	-	-	6 (22.2)	21 (77.8)	-	1.00
Technical requirement (possibility of web display)	-	-	5 (18.6)	22 (81.5)	-	1.00
Program modality (blended)	-	-	3 (11.1)	24 (88.9)	-	1.00
Learning assessment	-	1 (3.7)	5 (18.6)	20 (74.1)	1 (3.7)	0.96
CVI^*^						0.99
**Specific requirements for videos** There is harmony between colors, fonts, animations, vignettes and other digital resources	-	-	6 (22.2)	21 (77.8)	-	1.00
Good sound capture	-	-	1 (37.0)	17 (63.00)	9 (33.3)	1.00
Good image capture	-	-	7 (25.9)	20 (74.1)	-	1.00
Uses different formats: documentaries, animation, fiction, among others	-	-	6 (22.2)	21 (77.8)	-	1.00
The soundtrack is adequate to the theme	-	-	9 (33.3)	17 (63.0)	1 (3.7)	1.00
Approaches the contents in a logical, orderly and sequential manner	-	-	7 (25.0)	20 (74.1)	-	1.00
Presents language which is adequate to the level of the proposed education	-	-	6 (22.2)	21 (77.8)	-	1.00
Contains contextualized content consistent with the area and level of teaching proposed	-	-	6 (22.8)	21 (77.8)	-	1.00
Presents originality, variety and depth of the strategies addressed	-	-	9 (33.3)	18 (66.7)	-	1.00
Shows scientific rigor of the knowledge transmitted	-	-	5 (18.5)	22 (81.5)	-	1.00
Contemplates the diversity of accents, vocabularies and regional customs	1 (3.7)	2 (7.4)	8 (29.6)	11 (40.7)	5 (18.5)	0.86
There is identification by area of knowledge and curricular component(s)	-	-	9 (33.3)	15 (55.6)	3 (11.1)	1.00
There is identification by level(s) of education	-	1 (3.7)	10 (37.0)	12 (44.4)	4 (14.8)	0.96
Favors interdisciplinarity	-	3 (11.1)	15 (55.6)	9 (33.3)	-	0.89
Refers to the students’ everyday universe, from a perspective of training and citizenship	-	-	11 (40.7)	14 (51.9)	2 (7.4)	1.00
Presented in a playful, challenging and clear manner	-	-	6 (22.2)	21 (77.8)	-	1.00
The language aspects can stimulate the interest of students and professors	-	-	5 (18.5)	21 (77.8)	1 (3.7)	1.00
There is concern with aesthetics combined with content	-	-	8 (29.6)	19 (70.4)	-	1.00
Resorts to exemplification and analogies whenever possible	-	1 (3.7)	10 (37.0)	15 (55.6)	1 (3.7)	0.96
CVI^*^						0.98
**Interface** Free navigation	-	1 (3.7)	9 (33.3)	17 (63.0)	-	0.96
Clarity of information	-	1 (3.7)	5 (18.5)	21 (77.8)	-	0.96
Easy-to-locate information	-	-	9 (33.3)	18 (66.7)	-	1.00
Pertinence	-	-	9 (33.3)	18 (66.7)	-	1.00
Contextualization	-	-	8 (29.6)	19 (70.4)	-	1.00
Correction of the contents	-	1 (3.7)	9 (33.3)	13 (48.1)	4 (14.8)	0.96
Multiple windows	-	-	12 (44.4)	13 (48.1)	2 (7.4)	1.00
Interaction learning ease	-	-	10 (37.0)	16 (59.3)	1 (3.7)	1.00
Efficiency of use	-	-	8 (29.6)	18 (66.7)	1 (3.7)	1.00
Ease of return	-	-	10 (37.0)	17 (63.0)	-	1.00
Ergonomics	-	-	12 (44.4)	14 (51.9)	1 (3.7)	1.00
Aesthetics	-	-	8 (29.6)	19 (70.4)	-	1.00
Use of special marks	-	-	13 (48.1)	11 (40.7)	3 (11.1)	1.00
Presents audiovisual resources adequately	-	1 (3.7)	7 (25.9)	19 (70.4)	-	0.96
References	-	-	5 (18.5)	22 (81.5)	-	1.00
Interactivity	-	1 (3.7)	8 (29.6)	18 (66.7)	-	0.96
Navigation error management	-	-	12 (44.4)	6 (22.2)	9 (33.3)	1.00
Help for the users	2 (7.4)	1 (3.7)	9 (33.3)	12 (44.4)	3 (11.1)	0.87
Quality of the information	-	-	8 (29.6)	19 (70.4)	-	1.00
Robustness	-	-	10 (37.0)	16 (59.3)	1 (3.7)	1.00
Portability	-	-	11 (40.7)	10 (37.0)	6 (22.2)	1.00
Forecast updates	-	-	7 (25.9)	12 (44.4)	8 (29.6)	1.00
Reusability	-	-	9 (33.3)	18 (66.7)	-	1.00
CVI^*^						0.98

* CVI = Content Validity Index

A Gwet’s AC1 of 0.99 was obtained for the general and pedagogical requirements. In the case of the specific requirements for the videos and interface, Gwet’s AC1 was 0.97 in both. All requirements reached almost perfect agreement among the experts.

**Table 4:** Validation of the teaching program interface by experts in the Information Technology area (n = 7). Ribeirão Preto, SP, Brazil, 2020

Agreement Criterion	Strongly disagree	Disagree	Agree	Strongly agree	Don't know	CVI*
	n (%)	n (%)	n (%)	n (%)	n (%)	
**Interface** Free navigation	-	-	5 (71.4)	2 (28.6)	-	1.00
Clarity of information	-	-	3 (42.9)	3 (42.9)	1 (14.3)	1.00
Easy-to-locate information	-	-	3 (42.9)	3 (42.9)	1 (14.3)	1.00
Pertinence	-	1 (14.3)	3 (42.9)	3 (42.9)	-	0.86
Contextualization	-	-	5 (71.4)	2 (28.6)	-	1.00
Correction of the contents	-	-	2 (28.6)	3 (42.9)	2 (28.6)	1.00
Multiple windows	-	2 (28.6)	2 (28.6)	2 (28.6)	1 (14.3)	0.67
Interaction learning ease	-	1 (14.3)	4 (57.1)	2 (28.6)	-	0.86
Efficiency of use	-	-	6 (85.7)	1 (14.3)	-	1.00
Ease of return	-	-	4 (57.1)	3 (42.9)	-	1.00
Ergonomics	-	1 (14.3)	2 (28.6)	3 (42.9)	1 (14.3)	0.83
Aesthetics	-	1 (14.3)	2 (28.6)	3 (42.9)	1 (14.3)	0.83
Use of special marks	-	-	4 (57.1)	1 (14.3)	2 (28.6)	1.00
Presents audiovisual resources adequately	-	-	5 (71.4)	2 (28.6)	-	1.00
References	-	-	4 (57.1)	3 (42.9)	-	1.00
Interactivity	-	1 (14.3)	3 (42.9)	3 (42.9)	-	0.86
Navigation error management	-	-	2 (28.6)	3 (42.9)	2 (28.6)	1.00
Help for the users	-	-	4 (57.1)	2 (28.6)	1 (14.3)	1.00
Quality of the information	-	-	3 (42.9)	3 (42.9)	1 (14.3)	1.00
Robustness	-	-	3 (42.9)	3 (42.9)	1 (14.3)	1.00
Portability	-	1 (14.3)	1 (14.3)	5 (71.4)	-	0.86
Forecast updates	-	-	3 (42.9)	3 (42.9)	1 (14.3)	1.00
Reusability	-	-	3 (42.9)	3 (42.9)	1 (14.3)	1.00
CVI^*^						0.94

* CVI = Content Validity Index

Gwet’s AC1 was 0.89, achieving almost perfect agreement among the IT experts.

## Discussion

The blended learning program was elaborated and its theoretical content was organized into six short topics that covered the stages of the EP through infographics and videos considered attractive LO^(18)^.

The Nursing experts who participated in the validation of the general and pedagogical requirements, as well as of those specific to videos and interface, were mostly teachers and doctors; 14 (51.9%) of them had already developed at least one LO. Most of the IT experts worked with programming and technical support, which reflected in a sample qualified for the validation of the proposed teaching program.

Several adverse events have been associated with the AAA EI^(6,19-22)^. Therefore, the competences of health professionals (physicians, nurses and physiotherapists) who perform AAA have been evaluated and questioned regarding knowledge, skills and attitudes in a variety of tools, adapted in the context of the institutional policies where these professionals work^(23)^.

Validation research, like the present study, favors the appropriate selection of effective LO and methods for obtaining satisfactory results in the teaching and learning process. The development of teaching programs that foster critical thinking and clinical reasoning skills is essential and must offer learning opportunities that facilitate knowledge transfer^(24)^.

The teaching-learning process combined with the use of ICT, such as the use of videos with simulated clinical cases, contribute to the development of the clinical skills in Nursing, in an environment that reflects reality, linking the teaching method, the classroom and the practice^(25)^. Proposing teaching programs in the blended modality, with LO and face-to-face moments in clinical learning environments, with skills training and realistic simulation, seems to be something promising in the health area.

Blended learning poses challenges for students, institutions and educators. Unreal expectations, feelings of isolation, and time commitment are some of the challenges experienced by the students; the challenges for institutions and educators are mainly related to technical support and to the correct handling of the ICTs and LOs^(10)^.

The development of high quality blended education programs requires planning, time and selection of resources appropriate to the target audience and the educational objectives established. The results of this study showed that the pedagogical requirements and the proposed videos for the blended learning program on AAA were validated with an excellent CVI. However, it is noted that the students accustomed to a traditional format can still resist this new modality and not be interested in assuming responsibilities and controlling their own learning^(26)^.

The success of blended programs depends, not only on their validation and pedagogical quality, but also on how much the students are prepared to learn through this technology-mediated teaching^(27)^.

It is also necessary that the educators are prepared and take ownership of the ICT, with an emphasis on social networks. They must also develop skills with the VLE, especially Moodle and, finally, seek greater understanding of the human-computer interface and usability. It is essential to know the possibilities and limitations of the interface: the entire interaction of the student with the content, resources and LO takes place through it^(12)^. In this study, the interface requirements were validated by both Nursing and IT professionals, aiming at greater adequacy and success in the interaction between human and computer.

When there is a possibility of transition from a traditional program to the blended modality, the institution must offer support to the professors who have never taught online, with guidance from instructional designers and multimedia personnel, benefiting the leaders in their conceptions and design of innovative teaching programs^(26)^. In the past, a whiteboard and brush were the protagonists of this process. Nowadays, and in an irreversible way, it is the ICT that increasingly gain space and credibility^(9,25-26)^.

Direct instruction, with strict class schedules, is being replaced by videos and other attractive LOs, allowing the classroom experience to be better used in skills training activities and clinical simulations that contribute to the practice of students and nurses in the transition of virtual environments for assisting patients in a real environment^(28-29)^.

As limitations of this study, the absence of the usability evaluation of this teaching program from the student’s perspective is mentioned. It is suggested that future studies may evaluate its usability and its impacts on the teaching and learning process of AAA.

As for the benefits and advances in knowledge, a teaching program on AAA is available, which can be used both for the training of future nurses and for permanent education in health institutions. The mediation by technological devices of a digital culture for teaching and learning corroborates Vygotsky’s ideas^(11)^ and constitutes an innovative proposal.

## Conclusion

The blended learning program for aspiration of artificial airways was elaborated from a literature review, based on Vygotsky’s theoretical framework, and on the stages of the Nursing Process. It has been validated by Nursing and Information Technology experts. Almost perfect agreement was obtained on the general, pedagogical and specific requirements for videos and interface.
